# Nest wax triggers worker reproduction in the bumblebee *Bombus terrestris*

**DOI:** 10.1098/rsos.150599

**Published:** 2016-01-06

**Authors:** Ann-Marie Rottler-Hoermann, Stefan Schulz, Manfred Ayasse

**Affiliations:** 1 Institute of Evolutionary Ecology and Conservation Genomics, University of Ulm, Ulm, Germany; 2 Institute of Organic Chemistry, Technical University Braunschweig, Braunschweig, Germany

**Keywords:** conflict over male production, cuticular lipid, nest environment, social insect, wax scent

## Abstract

Social insects are well known for their high level of cooperation. Workers of the primitively eusocial bumblebee *Bombus terrestris* are able to produce male offspring in the presence of a queen. Nonetheless, they only compete for reproduction, in the so-called competition phase, when the workforce is large enough to support the rearing of reproductives. So far, little is known about the proximate mechanisms underlying the shift between altruism and selfish behaviour in bumblebee workers. In this study, we have examined the influence of chemical cues from the nest wax on the onset of worker reproduction. Chemical analyses of wax extracts have revealed that the patterns and amounts of cuticular lipids change considerably during colony development. These changes in wax scent mirror worker abundance and the presence of fertile workers. In bioassays with queen-right worker groups, wax affects the dominance behaviour and ovarian development of workers. When exposed to wax from a colony in competition phase, workers start to compete for reproduction. We suggest that wax scent enables workers to time their reproduction by providing essential information concerning the social condition of the colony.

## 1. Introduction

Thousands of social wasp, ant, bee and termite species are distributed worldwide and dominate many terrestrial habitats [1]. One key feature responsible for their great ecological and evolutionary success is their reproductive division of labour resulting in two kinds of female castes [2]. In most colonies of social Hymenoptera, reproduction is limited to one or a few individuals, the queens, whereas the majority of female nest-mates, the workers, remains sterile and focuses on tasks such as brood care, foraging, nest maintenance and defence [3]. As these colonies basically consist of mother and daughter groups, workers contribute indirectly to the next generation in ensuring the successful rearing of closely related individuals [4].

Despite their high level of cooperation, social insect colonies are not free of conflict [5]. Although unmated, most workers of Hymenopteran social insect species possess functional ovaries and are capable of laying haploid male-producing eggs [6,7]. Kin selection theory predicts that the asymmetric relatedness arising from haplodiploid sex determination leads to a conflict over male production, as workers contribute more genes to the next generation when reproducing instead of raising brothers [4,8]. In monogynous colonies with a singly inseminated queen, workers gain greater fitness by rearing sons (relatedness *r*=0.5) rather than brothers (*r*=0.25) and nephews (*r*=0.375). For an understanding of socio-biology in insects, we urgently need to reveal the proximate mechanisms that maintain reproductive bias in social insect colonies [9].

A pronounced conflict over male production is found in the bumblebee *Bombus terrestris*, which has been a model for studies on the regulation of reproduction in social insects for decades. In this species, workers are capable of laying eggs, despite queen control [10,11]. However, worker egg laying is limited to a later phase of colony development. In a newly established colony in spring, the monandrous founding queen is the only reproductive individual of the colony [12]. The social phase ends close to the end of the breeding season, when the colony starts to produce reproductives [2,13]. In the so-called competition phase, workers with developed ovaries become reproductively competitive and lay their own eggs. This stage of colony development is marked by egg policing and aggressive interactions among workers and between workers and the queen [14–18].

At least during the social phase of colony development, the presence of the queen prevents worker reproduction [19,20]. A primer pheromone produced by queens [21–23] and fertility signals on the cuticle surface mirroring the fecundity status of the queen [23–25] affect the ovarian development of young workers. However, queens are not capable of completely preventing ovarian development in workers; they merely delay it [26]. In the presence of a fertile queen (queen-right colony), 40% of the workers are capable of laying eggs by 10 days after their emergence [11], whereas under queenless conditions, a comparable development of worker ovaries is achieved within 6 days [10,27]. Nonetheless, workers with mature ovaries refrain from oviposition and resorb ripe eggs during the social phase of a queen-right colony [10,11,16]. Moreover, egg-laying workers even revert to sterility when transferred from competition-phase to social-phase colonies [28,29].

In terms of kin selection, hampering gyne production (*r*=0.75) is not in the workers’ interest [4]. As the colony reproduces only once, decisions on the optimal timing of reproduction events are crucial [30]. To maximize gyne production, workers should refrain from reproducing during the social phase when colony mortality is high [31,32] and instead focus on increasing worker numbers [33]. In the competition phase, when the largest possible workforce is available, workers can lay eggs without diminishing the number of gynes reared. Thus, workers can only increase their fitness gains by direct reproduction when they limit reproductive attempts to a later stage of colony development.

Several studies assumed that worker reproduction might be triggered by cues from the nest environment that indicate the social status of the colony. These cues might provide information, e.g. on nest population density [14,15,34–37], worker/larvae ratio [16] and the caste fate of diploid eggs [28,36–42]. Detailed knowledge of the developmental stage of the colony provided by cues from the nest environment might help workers to maximize their inclusive fitness. However, the respective information source remained unidentified so far.

In this study, we have focused on nest wax as a possible information source in *B. terrestris* colonies that might allow workers to optimize the timing of egg laying. Similar to honeybee wax [43–45], bumblebee wax contains a complex blend of cuticular lipids that have been secreted by the queen and workers [46]. These compounds have various functions in the inter-individual recognition of bumblebees, including fertility and task signalling [23,24,47,48]. Additionally, they serve as egg markings [49] and as markings at the nest entrance [50–52]. Associated with wax, these compounds provide information on colony identity and allow nest recognition [46]. Here, we have examined whether the function of wax scent can extend beyond nest-specific information. By chemically analysing the wax compound patterns of *B. terrestris* colonies at various stages of colony development, we have aimed to answer the following questions: (i) Does nest wax scent differ between social-phase and competition-phase colonies and thus provide information on the social status of the colony? (ii) Who contributes to the time-dependent changes in wax profiles? In addition, we have performed experiments with queen-right worker groups under variable environmental conditions to assess (iii) whether wax has an effect on worker reproductive behaviour and physiology.

## 2. Material and methods

### 2.1 Maintenance of bumblebees

Our study of the influence of nest wax scent on worker reproduction was performed with *B. terrestris* colonies of the laboratory-reared stock at the Institute of Experimental Ecology, University of Ulm, in 2009, 2010 and 2011. The founding queens of all colonies were descended from commercial colonies (Koppert Biological Systems, The Netherlands). They had been mated and hibernated for 10–12 weeks at a temperature of 6°C. After hibernation, colony founding was initiated in plastic rearing boxes (18×18×6.5 cm, Koppert Biological Systems, The Netherlands) and promoted by pairing two queens that competed for reproduction [53]. After the emergence of the first worker generation, the colonies were transferred into larger wooden boxes (39×16.5×16 cm). All colonies (source colonies for wax chemistry: *n*=4; for cuticle-surface extracts: *n*=3) and experimental subgroups (source colonies needed for behavioural assays: workers from *n*=8 colonies; social-phase wax from *n*=4 colonies; competition-phase wax from *n*=3 colonies; queens from *n*=20 social-phase colonies) were provided *ad libitum* with a 50% sugar solution of API-Invert^®^ (Südzucker AG, Germany) and fresh pollen (Koppert Biological Systems, The Netherlands). They were kept in constant darkness at a temperature of 27°C and a relative humidity of 70%. All observations and the collection of wax samples were performed under red light. To determine the onset of the competition phase, colonies were observed daily (three times for 30 min) for the occurrence of at least one of the following signs of worker reproduction: (i) worker oviposition, (ii) egg cell destruction, and (iii) oophagy [9,16,40]. We were able to observe egg laying by workers as direct evidence for the onset of competition in all colonies that were included in the experiments. The onset of competition in our colonies occurred 31±1 days after the first worker emerged (matching the observations of Duchateau & Velthuis [16] and Alaux *et al.* [38]).

### 2.2 Collection of wax extracts and cuticle-surface samples

For chemical analyses, we collected wax samples from four *B. terrestris* colonies (*n*=20 wax samples from each colony) at various stages of colony development (colony age is given in days after the first worker emerges and colony size is given in the number of workers present in the colony): twice during the social phase at (i) a colony age of 15 days and a colony size of 10–15 workers (social phase 1) and at (ii) 25 days with about 50 workers (social phase 2), and twice during the competition phase at (iii) 35 days (in the case of our colonies, 3–5 days after onset of competition) with about 100 workers (competition phase 1) and at (iv) 45 days with about 150 workers before the first reproductives emerged (competition phase 2). For each sample, an average of 3 mg of wax was taken from various wax types, including honey pots (*n*=2 from each colony at each sampling point) and brood cells (*n*=3 from each colony at each sampling point), which had been built recently (wax that is older than a few days darkens considerably and was excluded from our analyses). Wax pieces were weighed, transferred to glass vials (1.5 ml) and extracted in 200 μl pentane (Merck, Germany) at room temperature for 30 min, while being vortexed twice. Samples were kept frozen (minus 40°C) for at least 1 h to allow sedimentation of insoluble, non-volatile wax components [54]. For gas chromatographic analyses, 20 μl aliquots of the wax extracts were diluted in pentane (1:3). Additionally, cuticle-surface extracts of the abdomen of (i) sterile workers (*n*=31), (ii) fertile workers (*n*=50) and (iii) breeding queens (*n*=9) were included in the analyses to examine the origin of wax profile differences during colony development. Sterile workers with undeveloped ovaries (undifferentiated oocytes <0.18 mm [11]) and fertile workers with mature ovaries (at least one oocyte fully developed [11]) were taken from five source colonies (during social phase: 25 sterile workers, five fertile workers; during competition phase: six sterile workers, 45 fertile workers). The queens were obtained from nine social-phase colonies with 15 to 20 workers. Individual workers and queens were killed and stored at −40°C until dissection. Cuticle-surface extracts were obtained by rinsing an individual abdomen for 30 s in pentane (worker: 0.5 ml; queen: 1 ml; Uvasol, 99.5%, Merck, Germany; extraction method following [24]). After extraction, the extract was transferred to a new glass vial (1.5 ml). All samples were kept frozen at −40°C until analysed. *n*-C10 (1 μg) was added as an internal standard to wax and cuticle-surface samples to quantify the absolute amounts of compounds extracted from wax and cuticle surfaces. To determine putative variations in the profile (same compounds present in the samples but differences in compound ratios) of cuticular lipids in wax and to determine similarities to cuticle-surface profiles of workers and queens, we calculated the relative amount (%) of each compound in respect to the total concentration of the whole bouquet (76 compounds included in the analyses).

### 2.3 Chemical analyses

The chemical analyses of all extracts were performed on an Agilent 7890 A Series gas chromatograph (Agilent Technologies, Germany) with a flame ionization detector (detector temperature: 310°C) and a DB-5 capillary column (30 m × 0.25 mm inner diameter, J&W), with the carrier gas hydrogen (constant flow 2.0 ml min^−1^). The split valve opened 1 min after splitless injection (injector temperature 310°C) of 1 μl of the samples; the oven temperature increased by 10°C min^−1^ from 50°C to 310°C. The identification of 76 compounds was based on the results of former gas chromatographic mass spectrometric analyses [46] and the comparison of mass spectra (gas chromatography mass spectrometry, HP 6890 Series GC System and HP 5973 Mass Selective Detector (Hewlett-Packard Company, Wilmington, USA)) and gas chromatographic retention indices with those of authentic references, of literature data from the *B. terrestris* semicochemical complex [24,25], of mass spectral libraries (Nist08 database, Wiley 7 database) and of non-public internal databases. The absolute and relative amounts of these compounds were measured with Agilent ChemStation software (Agilent Technologies, Germany).

### 2.4 Behavioural assays on worker reproduction

To determine the effect of cues from the nest environment on bumblebee social behaviour, we examined the interactions and physiology of queen-right worker groups under one of the following conditions: 10 newly emerged workers (less than 6 h old; randomly taken from eight source colonies that had entered the competition phase 5–10 days before) were put together with a breeding queen (taken from social-phase colonies where 5–15 workers had already emerged) and were provided (i) with no nest environment (empty), (ii) with a comb containing brood from social-phase colonies (social-phase nest; five source colonies), (iii) with 5 g of wax from social-phase colonies (social-phase wax; four source colonies), and (iv) with 5 g of wax from competition-phase colonies (competition-phase wax; three source colonies; each treatment: *n*=5). In all groups, workers were not related to the queen and both queens and workers were not exposed to wax or nest from their original colonies. Since ovarian development and dominance interactions of newly emerged workers were shown to be unaffected by relatedness/unrelatedness [55], the random grouping of newly emerged workers from different source colonies was possible without risking nest-mate/non-nest-mate conflicts. In creating artificial groups that resembled the queen–worker ratio of early social-phase colonies, we were able to examine the direct effect of different treatments on worker behaviour and ovarian development in groups with otherwise identical preconditions (workers of the same age and random genetic heritage).

All groups were maintained in plastic rearing boxes (18×18×6.5 cm, Koppert Biological Systems, Berkel en Rodenrijs, The Netherlands) and were supplied with sugar solution and fresh pollen *ad libitum* for 6 days. During this time, the behaviour of each group was video-taped every 48 and 72 h for 15 min (Canon PowerShot SD1100 IS Digital ELPH, Canon Inc., USA). The videos were analysed by using the observation software The Observer v. 5.0 (Noldus Information Technology, The Netherlands). We examined all direct interactions between two workers or the queen and a worker and assigned the outcome of the interaction to one of these three categories (and their associated behaviours): (i) neutral interaction (antennating), (ii) aggressive interaction (threatening, i.e. front leg movements and inclination of mandibles, darting or head butting, biting and stinging attempts), and (iii) defensive interaction (backing-off). In the case that a threatening event was followed by an immediate attack these behaviours were counted as one aggressive interaction. From these data, we determined the total number of interactions per minute within the group (sum of neutral and agonistic interactions), and the number of neutral interactions, aggressive interactions and defensive interactions of which the latter two are associated with the establishment of dominance in bumblebee colonies. After 6 days at the end of the experiment, the workers were killed by being frozen at −20°C and were dissected (under a stereo microscope) in distilled water to determine the developmental status of their ovaries. We classified three developmental stages of the ovaries [11]: (i) undeveloped ovaries (undifferentiated oocytes <0.18 mm), (ii) developing ovaries (initiated vitellogenesis, terminal oocytes>0.18 mm, trophocytes still visible), and (iii) mature ovaries (at least one oocyte fully developed).

### 2.5 Statistical analyses

All data obtained from the chemical analyses and behavioural assays were documented in Excel 2007 (Microsoft), analysed and illustrated in SigmaStat v. 3.5 (Systat Software Inc.), SPSS v. 16.0 for Windows (SPSS Inc.), PRIMER v. 6.1.6 (Primer-E Ltd., Plymouth), R (v. 3.2.2) and Corel draw x5 (Corel Corporation). The level of significance was defined at *α*=0.05 for all statistical analyses.

The statistical analyses of the chemical composition of 80 wax scent samples and 90 bumblebee cuticle extracts were performed with the relative amounts of 76 peaks with a concentration more than 0.01% of the total substance amount that occurred in all samples. For multivariate analyses on changes in the wax scent pattern during colony development, we performed a square root transformation of the relative amounts of compounds to down-weigh the contributions of quantitatively dominant compounds to the similarities calculated between the samples. Two one-way analyses of similarities (ANOSIM with 999 permutations) based on the Bray–Curtis similarity index were performed to determine similarities/dissimilarities between wax from social-phase and competition-phase colonies and the cuticle-surface profiles of workers and queens. *R*-values between 0 and 1 specified the level of similarity, whereas *R*=0 implied no difference between the groups and *R*=1 represented larger similarities within the group than between the groups [56]. Non-metric multidimensional scaling (NMDS) was employed to visualize differences and similarities of the datasets. Stress values indicated the level of reliability of the two-dimensional representation of sample type relationships (stress values less than 0.1: good quality of regression). To determine the compounds that mainly contributed to differences between social-phase and competition-phase wax (similarity percentage), we performed a SIMPER analysis. Because of the numerous compounds found in the odour bouquets, we focused on compounds that contribute more than 1.8% to the average Bray–Curtis similarity and dissimilarity of the groups. To demonstrate that changes in wax scent pattern during colony development occur independent from colony identity, we examined the effect of colony identity (nest A, B, C, D), colony age (social phase 1, social phase 2, competition phase 1, competition phase 2) and wax type (honey pot and brood cell) on the relative compound amounts by computing generalized linear mixed models (GLMMs; repeated measure approach including colony identity as random factor).

Average Bray–Curtis similarity values between each cuticle-surface profiles and all wax profiles from social-phase and competition-phase colonies were used to compare the contribution of queens, sterile workers and fertile workers to the nest wax scent at various stages of colony development. Further comparisons between the relative amounts of compounds of wax samples and cuticle-surface samples of (i) sterile workers, (ii) fertile workers, and (iii) breeding queens were conducted by using SIMPER analyses and Kruskal–Wallis nonparametric tests followed by *post hoc* Tukey tests.

For the statistical analysis of the behavioural results, we computed multivariate general linear models (GLMs) to test the effect of a differing nest environment (i.e. empty, social-phase nest, social-phase wax and competition-phase wax) on (i) the total number of interactions (min^−1^) (GLM, quasi-Poisson distribution; R package lme4), (ii) the number of aggressive interactions (min^−1^) (GLM, quasi-Poisson distribution), (iii) the number of defensive interactions (min^−1^) (GLM, quasi-Poisson distribution), (iv) the number of workers with undeveloped ovaries (GLM, quasi-Poisson distribution), (v) the number of workers with developing ovaries within a group (GLM, quasi-Poisson distribution), (vi) the presence or the absence of workers with mature ovaries (GLM, binomial distribution (link = logit); owing to zero-inflated probability distribution of groups with workers with mature ovaries), all (i) to (vi) within each worker group (*n*=5 for each of the four treatments).

## 3. Results

### 3.1 Effect of colony development on wax scent profiles and concentration

**Figure 1.**
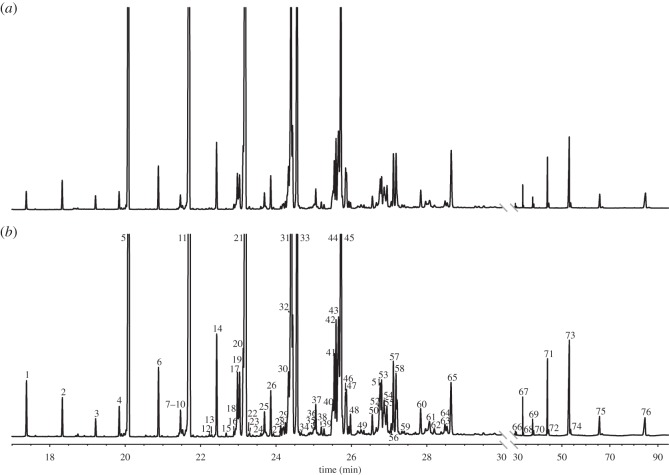
Gas chromatogram of wax extracts taken from a bumblebee colony during (*a*) the social phase and (*b*) the competition phase of colony development (numbers indicate peak identity, table 1).

**Figure 2.**
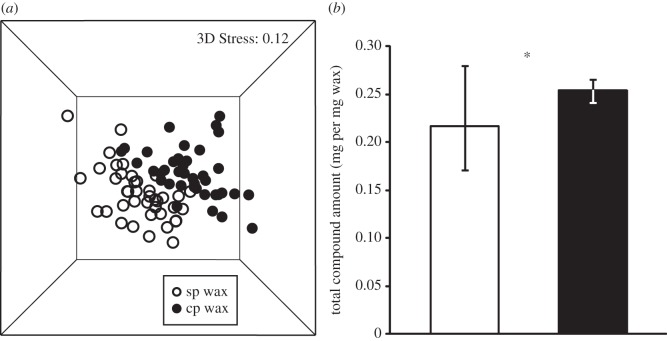
Differences in (*a*) wax scent profiles and (*b*) total amounts of wax compounds from social-phase (sp) and competition-phase (cp) colonies. (*a*) Based on relative amounts of compounds, wax scent profiles differed between sp and cp colonies (NMDS, Bray–Curtis similarity measure; 3D Stress 0.12; sp, open circles; cp, filled circles). (*b*) Cp wax contained significantly higher absolute amounts of compounds than sp wax (median, 25th and 75th percentile; sp, white bar; cp, black bar; *U*-test: *U*=1413, *n*=40, *p=0.047).

In all wax samples examined, we found 76 compounds that have been previously described to form the scent pattern of *B. terrestris* wax [46] and the scent pattern on the cuticle surface of workers and queens [24]. The relative amount of these compounds (table 1) and the resulting wax profile (social-phase wax profiles *n*=40, competition-phase wax profiles *n*=40; ANOSIM: *R*=0.292, *p*=0.001; NMDS: 3D Stress 0.12, figure 2*a*) changed considerably during colony development (social-phase and competition-phase wax chromatograms, figure 1). Most changes in relative compound amounts with colony age occurred independently from colony identity and wax type (electronic supplementary material, appendix A). Social-phase and competition-phase wax profiles could be distinguished by various substances, i.e. by odd-numbered alkanes tricosane (*n*-C23), pentacosane (*n*-C25), heptacosane (*n*-C27) and nonacosane (*n*-C29), by the long-chained alkenes (*Z*)-11-nonacosene, hentriacontene and tritriacontene, by the alkadienes hentriacontadiene and tritriacontadiene, by the ethyl ester ethyl hexadecanoate, by the wax esters icosyl octadecenoate, hexacosyl octadecenoate and triacontyl octadecenoate, by the aldehydes triacontanal and dotriacontanal and by the ketone 2-nonacosanone (relative substance amounts, *U*-test statistics and SIMPER % of all compounds, table 1).

**Table 1.** Comparison of the relative amounts of 76 compounds examined in wax extracts from social-phase (sp) and competition-phase (cp) colonies (median, 25th and 75th percentile; *U*-test statistics, SIMPER contributing % of these compounds). Compounds are sorted by substance class (italics) and the numbers given with the compound names encode peak identity (figure 1). Rows marked in bold indicate compounds that had a high contribution to differences between social-phase and competition-phase wax.

compounds	sp wax (*n*=40)	cp wax (*n*=40)	*U*	*p*-value	SIMPER %
*alkanes*					
henicosane (2)	0.33 (0.31, 0.41)	0.46 (0.40, 0.55)	297.00	<0.001	1.71
docosane (3)	0.15 (0.14, 0.16)	0.18 (0.16, 0.19)	345.00	<0.001	0.58
**tricosane** (**5**)	**10.40** (**9.96**, **11.03**)	**12.11** (**10.44**, **12.41**)	**340.00**	**<0.001**	**3.65**
tetracosane (6)	0.47 (0.45, 0.49)	0.48 (0.44, 0.49)	730.00	(n.s.) 0.501	0.38
pentacosane (11)	15.95 (15.39, 16.40)	16.30 (15.29, 16.84)	705.00	(n.s.) 0.361	1.88
hexacosane (14)	0.75 (0.74, 0.77)	0.71 (0.69, 0.73)	226.00	<0.001	0.40
heptacosane (21)	15.85 (15.24, 16.26)	14.92 (14.52, 15.37)	289.00	<0.001	1.87
octacosane (26)	0.37 (0.35, 0.39)	0.34 (0.32, 0.35)	233.00	<0.001	0.72
**nonacosane** (**33**)	**6.88** (**6.59**, **7.50**)	**6.24** (**5.88**, **6.75**)	**301.00**	**<0.001**	**2.71**
triacontane (38)	0.08 (0.07, 0.10)	0.07 (0.06, 0.08)	501.00	0.004	0.88
hentriacontane (47)	0.48 (0.43, 0.55)	0.45 (0.36, 0.52)	604.00	(n.s.) 0.059	1.28
tritriacontane (56)	0.07 (0.06, 0.08)	0.11 (0.09, 0.12)	264.00	<0.001	0.92
*alkenes*					
(Z)-9-tricosene (4)	0.00 (0.00, 0.00)	0.00 (0.00, 0.00)	609.00	(n.s.) 0.066	0.03
(Z)-10-pentacosene (7)	0.03 (0.02, 0.03)	0.03 (0.03, 0.04)	257.00	<0.001	0.73
(Z)-9-pentacosene (8)	0.16 (0.14, 0.17)	0.16 (0.14, 0.17)	764.00	(n.s.) 0.729	0.80
(Z)-8-pentacosene (9)	0.02 (0.02, 0.03)	0.03 (0.02, 0.03)	501.00	0.004	0.84
(Z)-7-pentacosene (10)	0.05 (0.05, 0.06)	0.05 (0.05, 0.05)	731.00	(n.s.) 0.507	0.32
(Z)-11- and (Z)-10-heptacosene (16)	0.06 (0.05, 0.07)	0.07 (0.06, 0.09)	472.00	0.002	0.70
(z)-9-heptacosene (17)	0.47 (0.44, 0.49)	0.48 (0.43, 0.52)	706.00	(n.s.) 0.366	0.82
(Z)-8-heptacosene (18)	0.09 (0.07, 0.09)	0.10 (0.09, 0.11)	448.00	0.001	0.71
(Z)-7-heptacosene (19)	0.41 (0.37, 0.46)	0.41 (0.37, 0.46)	789.00	(n.s.) 0.916	0.96
octacosene^a^ (25)	0.23 (0.22, 0.34)	0.22 (0.21, 0.25)	580.00	0.034	1.23
(Z)-11-nonacosene (30)	0.79 (0.73, 0.87)	0.92 (0.84, 1.07)	306.00	<0.001	1.88
(Z)-9-nonacosene (31)	5.17 (4.95, 5.29)	4.94 (4.57, 5.37)	641.00	(n.s.) 0.126	1.79
(Z)-7-nonacosene (32)	0.84 (0.69, 0.95)	0.79 (0.73, 0.89)	686.00	(n.s.) 0.273	1.67
triacontene^a^ (35)	0.04 (0.03, 0.05)	0.05 (0.04, 0.06)	461.00	0.001	0.58
triacontene^a^ (36)	0.07 (0.06, 0.08)	0.08 (0.07, 0.10)	354.00	<0.001	0.75
triacontene^a^ (37)	0.24 (0.23, 0.26)	0.21 (0.19, 0.23)	299.00	<0.001	0.78
hentriacontene^a^ (42)	0.64 (0.61, 0.66)	0.72 (0.68, 0.75)	246.00	<0.001	0.73
hentriacontene^a^ (43)	0.83 (0.80, 0.88)	0.81 (0.75, 0.88)	621.00	(n.s.) 0.085	0.75
**hentriacontene**^a^ (**44**)	**1.72** (**1.60**, **1.82**)	**1.96** (**1.90**, **2.37**)	**242.00**	**<0.001**	**2.38**
**hentriacontene**^a^ (**45**)	**5.51** (**5.11**, **5.72**)	**4.67** (**4.21**, **4.95**)	**140.00**	**<0.001**	**3.40**
tritriacontene^a^ (54)	0.41 (0.35, 0.45)	0.47 (0.41, 0.55)	477.00	0.002	1.97
tritriacontene^a^ (55)	0.32 (0.25, 0.37)	0.23 (0.17, 0.28)	292.00	<0.001	1.60
pentatriacontene^a^ (63)	0.08 (0.07, 0.10)	0.11 (0.08, 0.13)	402.00	<0.001	1.06
*alkadienes*					
nonacosadiene^a^ (28)	0.04 (0.04, 0.05)	0.05 (0.05, 0.06)	458.00	0.001	0.5
nonacosadiene^a^ (29)	0.15 (0.12, 0.16)	0.16 (0.15, 0.17)	517.00	0.006	0.57
triacontadiene^a^ (34)	0.06 (0.05, 0.08)	0.08 (0.07, 0.09)	445.00	0.001	0.87
hentriacontadiene^a^ (40)	0.21 (0.14, 0.28)	0.22 (0.17, 0.28)	782.00	(n.s.) 0.862	1.86
hentriacontadiene^a^ (41)	0.21 (0.19, 0.24)	0.29 (0.26, 0.32)	228.00	<0.001	1.60
**tritriacontadiene**^a^ (**51**)	**0.31** (**0.16**, **0.42**)	**0.18** (**0.13**, **0.27**)	**592.00**	**0.045**	**2.68**
**tritriacontadiene**^a^ (**52**)	**0.36** (**0.32**, **0.42**)	**0.58** (**0.51**, **0.69**)	**210.00**	**<0.001**	**2.51**
tritriacontadiene^a^ (53)	0.38 (0.35, 0.42)	0.43 (0.39, 0.49)	446.00	<0.001	0.85
pentatriacontadiene^a^ (61)	0.18 (0.15, 0.23)	0.20 (0.17, 0.23)	661.00	(n.s.) 0.181	0.90
*methylated alkanes*					
11-methylpentacosane, 9-methylpentacosane (12)	0.00 (0.00, 0.01)	0.01 (0.00, 0.01)	753.00	(n.s.) 0.651	0.53
5-methylpentacosane (13)	0.01 (0.01, 0.02)	0.01 (0.01, 0.02)	601.00	(n.s.) 0.056	0.37
13-methylheptacosane (22)	0.01 (0.01, 0.02)	0.01 (0.01, 0.01)	349.00	<0.001	0.44
11-methylheptacosane (23)	0.03 (0.02, 0.03)	0.03 (0.02, 0.03)	725.00	(n.s.) 0.470	0.44
3-methylheptacosane (24)	0.03 (0.02, 0.03)	0.03 (0.02, 0.03)	656.00	(n.s.) 0.166	0.79
*ethylesters*					
**ethyl hexadecanoate** (**1**)	**0.44** (**0.23**, **0.61**)	**0.24** (**0.14**, **0.36**)	**493.00**	**0.003**	**3.46**
ethyl octacosanoate (50)	0.01 (0.00, 0.02)	0.01 (0.01, 0.02)	770.00	(n.s.) 0.773	0.79
ethyl triacontanoate (60)	0.28 (0.24, 0.32)	0.23 (0.19, 0.25)	375.00	<0.001	1.16
*wax esters*					
hexadecyl tetradecanoate (49)	0.07 (0.06, 0.09)	0.10 (0.08, 0.13)	369.00	<0.001	1.00
icosyl hexadecanoate (66)	0.07 (0.05, 0.09)	0.06 (0.05, 0.07)	606.00	(n.s.) 0.062	0.71
**icosyl octadecenoate**^a^ (**67**)	**0.60** (**0.47**, **0.81**)	**0.58** (**0.47**, **0.90**)	**785.00**	(**n.s.**)**0.885**	**2.15**
docosyl hexadecanoate, icosyl octadecanoate (68)	0.12 (0.10, 0.15)	0.12 (0.10, 0.17)	748.00	(n.s.) 0.617	1.02
docosyl octadecenoate^a^ (69)	0.46 (0.41, 0.53)	0.40 (0.33, 0.54)	617.00	(n.s.) 0.078	1.30
tetracosyl hexadecanoate, docosyl octadecanoate (70)	0.29 (0.24, 0.36)	0.27 (0.23, 0.32)	683.00	0.260	1.33
tetracosyl octadecenoate^a^ (71)	4.35 (4.23, 4.55)	4.49 (4.36, 4.67)	525.00	0.008	1.16
hexacosyl hexadecanoate, tetracosyl octadecanoate (72)	0.40 (0.33, 0.48)	0.40 (0.34, 0.47)	799.00	(n.s.) 0.992	1.45
**hexacosyl octadecenoate**^a^ (**73**)	**11.07** (**10.60**, **11.60**)	**11.86** (**11.53**, **12.40**)	**339.00**	**<0.001**	**2.38**
octacosyl hexadecanoate, hexacosyl octadecanoate (74)	0.51 (0.41, 0.60)	0.45 (0.38, 0.51)	540.00	0.012	1.53
octacosyl octadecenoate^a^ (75)	2.27 (2.01, 2.46)	2.37 (2.17, 2.58)	604.00	(n.s.) 0.059	1.76
**triacontyl octadecenoate**^a^ (**76**)	**3.89** (**2.95**, **3.98**)	**3.41** (**2.88**, **4.11**)	**785.00**	**n.s.0.885**	**4.46**
*aldehydes*					
triacontanal (58)	0.50 (0.43, 0.58)	0.49 (0.37, 0.58)	730.00	(n.s.) 0.501	1.80
**dotriacontanal** (**65**)	**0.69** (**0.54**, **0.76**)	**0.53** (**0.40**, **0.70**)	**503.00**	**0.004**	**2.38**
*ketones*					
**2-nonacosanone** (**48**)	**0.15** (**0.07**, **0.25**)	**0.10** (**0.05**, **0.14**)	**553.00**	**0.017**	**2.18**
2-triacontanone (59)	0.04 (0.03, 0.06)	0.08 (0.06, 0.11)	354.00	<0.001	1.28
*acetates*					
tetracosyl acetate (27)	0.02 (0.02, 0.03)	0.03 (0.02, 0.03)	585.00	0.039	0.48
hexacosyl acetate (39)	0.06 (0.05, 0.07)	0.06 (0.06, 0.08)	517.00	0.006	0.47
*unidentified*					
unidentified (15)	0.03 (0.01, 0.05)	0.02 (0.02, 0.03)	729.00	(n.s.) 0.494	1.08
unidentified (20)	0.69 (0.63, 0.73)	0.61 (0.55, 0.68)	535.00	0.011	1.64
unidentified (46)	0.53 (0.46, 0.64)	0.47 (0.36, 0.52)	417.00	<0.001	1.63
unidentified (57)	0.73 (0.66, 0.79)	0.61 (0.51, 0.67)	360.00	<0.001	1.68
unidentified (62)	0.01 (0.01, 0.02)	0.01 (0.01, 0.02)	771.00	0.780	0.61
unidentified (64)	0.10 (0.08, 0.11)	0.08 (0.06, 0.10)	489.00	0.003	0.77

^a^Unknown double bond position.

In addition to changes in the relative amounts of compounds, we also found changes in the total compound amounts during colony development. The compound concentration (mg extracted from 1 mg of wax, median; 25th and 75th percentile) was higher in competition-phase than in social-phase wax (social-phase wax (0.22; 0.17, 0.28), competition-phase wax (0.25; 0.24, 0.27) *U*-test: *U*=1413, *n*=40, *p=0.047; figure 2*b*).

### 3.2 Origin of changes in wax scent profiles

**Figure 3.**
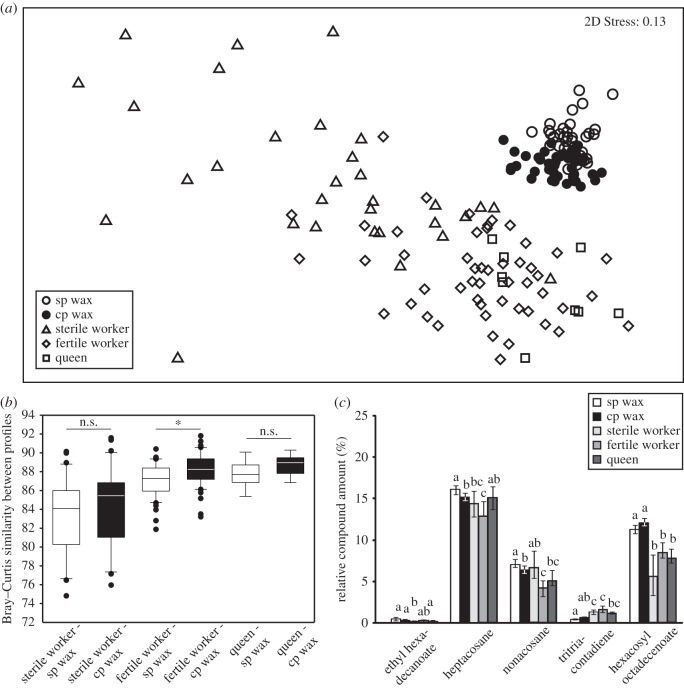
(*a*) Scent pattern differences and (*b*) similarities of social-phase (sp) wax, competition-phase (cp) wax, and cuticle-surfaces of queens, sterile workers and fertile workers, and (*c*) relative amounts of linear alkanes and oxygenated compounds that contributed to the pattern variations. (*a*) Wax scent and cuticle-surface bouquets differed in the relative composition of compounds (NMDS, Bray–Curtis similarity measure; 2D Stress, 0.13). (*b*) Comparison of Bray–Curtis similarities between cuticle-surface profiles of queens, sterile and fertile workers and wax profiles from sp and cp colonies (*U*-test: sterile worker-sp wax against sterile worker-cp wax, *U*=559, *n*=31, *p*=0.272; fertile worker-sp wax against fertile worker-cp wax, *U*=1709, *n*=50, *p=0.002; queen-sp wax against queen-cp wax, *U*=66, *n*=9, *p*=0.093). (*c*) The relative amounts of linear alkanes and oxygenated compounds that contributed to the pattern variations differed between sp wax, cp wax and female cuticle-surface profiles (median, 25th and 75th percentile; Kruskal–Wallis with *post hoc* Dunn’s tests (electronic supplementary material, appendix B): different letters indicate significant differences).

To determine the source of changes in the wax profile during colony development, we compared social-phase and competition-phase wax with cuticle-surface blends of queens and of sterile and fertile workers. In an NMDS plot, the bouquets of the various sample types were well separated (social-phase wax profiles *n*=40, competition-phase wax profiles *n*=40, queen profiles *n*=9, sterile worker profiles *n*=31, fertile worker profiles *n*=50; ANOSIM: global *R*=0.575, *p*<0.001, all pairwise comparisons: *p*=0.01; NMDS: 2D Stress 0.13, figure 3*a*). Comparing the average Bray–Curtis similarities between each surface profile (queens, sterile workers and fertile workers) and all wax profiles, we found that, irrespective of the developmental stage of the colony, the wax scent was more similar to queens (median, 25th and 75th percentile; 88.34, 87.58 and 89.04, *n*=9) and fertile workers (87.76, 86.63 and 88.88, *n*=50) than to sterile workers (84.77, 80.77 and 86.38; Kruskal–Wallis test, *H*_2_=19.06, *p*<0.001, *post hoc* Dunn’s method: *p*<0.05 for average Bray–Curtis values (wax, sterile worker) versus (wax, fertile worker), and (wax, sterile worker) versus (wax, queen); *p*>0.05 for (wax, fertile worker) versus (wax, queen)). Whereas the average similarity values between sterile worker profiles and wax (*U*-test statistics, average Bray–Curtis values (sterile worker, social-phase wax) versus (sterile worker, competition-phase wax), *U*=559, *n*=31, *p*=0.272) and queen profiles and wax (*U*-test statistics, average Bray–Curtis values (queen, social-phase wax) versus (queen, competition-phase wax), *U*=66, *n*=9, *p*=0.093; figure 3*b*) were not affected by colony phase, the wax scent of competition-phase colonies was more similar to the surface profiles of fertile workers (*U*-test statistics, average Bray–Curtis values (fertile worker, social-phase wax) versus (fertile worker, competition-phase wax), *U*=1709, *n*=50, *p*=0.002; figure 3*b*). This shift of the compound composition in competition-phase wax towards profiles of fertile workers also became apparent when comparing the relative amounts of the compounds that mainly contributed to the separation of social-phase and competition-phase wax (list of relative amounts of all compounds, Kruskal–Wallis test statistics and *post hoc* Dunn’s test, see the electronic supplementary material, appendix B). Substances, e.g. heptacosane, nonacosane and ethyl hexadecanoate, which were found in lower relative amounts in fertile workers and queens than in sterile workers, were also decreased in competition-phase compared with social-phase wax profiles (figure 3*c*). Other substances that mainly contributed to the discrimination of social-phase and competition-phase wax, such as tritriacontadiene and hexacosyl octadecenoate, and that were present in competition-phase wax in higher amounts tended to be higher in fertile than in sterile worker surface profiles.

### 3.3 Effect of wax on worker behaviour and physiology

**Figure 4.**
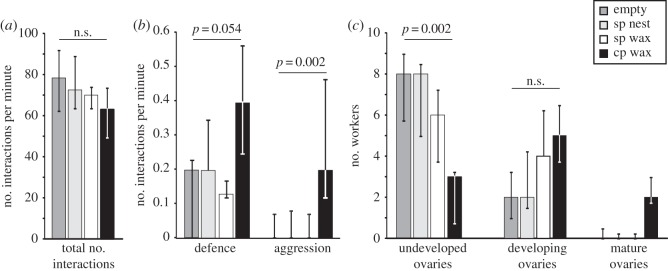
Differences in (*a*) the total number of interactions per min, (*b*) the number of aggressive and defensive interactions and (*c*) the number of workers with different ovarian development within worker groups exposed to various treatments (empty, social-phase (sp) nest, sp wax and competition-phase (cp) wax). (*a*) The treatment had no effect on the number of interactions per minute in queen-right worker groups (quasi-Poisson GLM, *F*_3,16_=0.981, *p*=0.423). (*b*) More aggressive and defensive interactions per minute occurred in groups exposed to cp wax (quasi-Poisson GLM aggression, *F*_3,16_=7.410, *p*=0.002; defence, *F*_3,16_=3.150, *p*=0.054). (*c*) In the cp wax groups, the number of workers with undeveloped ovaries was decreased (quasi-Poisson GLM: *F*_3,16_=7.828, *p*=0.002) and the presence of workers with mature ovaries was increased (binomial GLM: *χ*_3,16_=15.012, *p*=0.008). Bars represent the median, 25th and 75th percentiles.

In our bioassay, the nest environment (treatment: (i) empty, (ii) social-phase nest, (iii) social-phase wax and (iv) competition-phase wax; *n*=5 for each treatment) had a pronounced effect on behavioural interactions between workers and on the ovarian development of workers (figure 4). Whereas the total amount of interactions per minute was not affected by the treatment (quasi-Poisson GLM: *F*_3,16_=0.981, *p*=0.423; figure 4*a*), the number of aggressive interactions (quasi-Poisson GLM: *F*_3,16_=7.410, *p*=0.002; figure 4*b*) and the number of defensive interactions (quasi-Poisson GLM: *F*_3,16_=3.150, *p*=0.054; figure 4*b*) differed between different treatments. Aggressive and defensive interactions occurred more frequently in worker groups that encountered competition-phase wax than in any other treatment (figure 4*b*). Of the aggressive interactions, biting and stinging attempts only occurred in groups exposed to competition-phase wax, whereas darting behaviour was observed in all treatments. In all treatments, we recorded no aggressive and some defensive reactions towards the queen (% of defensive interactions towards queen, median, 25th and 75th percentile: 9.09, 0.00 and 35.00). In return, the queens were not observed to attack or back-off from workers.

Apart from its influence on worker behaviour, the treatment also had an effect on the number of workers with undeveloped ovaries (quasi-Poisson GLM: *F*_3,16_=7.828, *p*=0.002; figure 4*c*) and on the presence/absence of workers with mature ovaries (binomial GLM: *χ*_3,16_=15.012, *p*=0.008, figure 4*c*). In groups with competition-phase wax compared with the other treatments, the ratio of undeveloped ovaries was lower, whereas the presence of workers with mature ovaries was increased (figure 4*c*). Treatment had no significant effect on the number of workers with developing ovaries (quasi-Poisson GLM: *F*_3,16_=2.568, *p*=0.091; figure 4*c*).

## 4. Discussion

### 4.1 Effect of colony development on wax scent profiles and concentration

Our chemical analyses of wax scent revealed that the concentration and the relative composition of cuticular lipid compounds in wax changed dramatically during colony development. Wax from older colonies contained an increased amount of cuticular lipids. This increase might be caused by a constant reutilization of wax that can result in an accumulation of cuticular compounds over time. The presence of a higher number of workers could additionally contribute to the observed increase in wax scent during the later stages of colony development [16]. In mirroring the age of the colony and/or the number of workers present in the colony, wax lipid compound concentration could function as a cue that influences the onset of worker reproduction. Previous studies have shown a link between worker number and the timing of worker reproduction in bumblebee colonies ([14,15,35], but see [38]). Furthermore, an artificial increase in the number of workers has been shown to elicit an earlier onset of the competition phase [36]. So far, the proximate mechanism linking worker number and worker egg laying has remained unknown. It has been suggested that the queen might be unable to prevent workers from reproducing in larger colonies, even at her maximum level of activity [14,15]. However, this hypothesis has always faced the problem that, in particular, the workers that have most of the contacts with the queen during all phases of colony development become the principal egg layers in the competition phase [15].

Independent of changes of the queen’s influence with increasing colony size, the number of workers has been suggested to be crucial for the successful rearing of female reproductives [30]. To maximize gyne production, workers should refrain from reproducing at an early stage of colony development when colony mortality is high [31,32]. Workers can lay eggs without diminishing the number of gynes reared only when the largest possible workforce is available. Thus, information on the actual number of workers can be essential for the timing of reproductive decisions. Our chemical analyses of nest wax indicate that workers might gain the respective information concerning colony size from the concentration of cuticular lipids in the nest wax. This would be a remarkable way for workers to assess the size of the workforce independently of encounter rates with other colony members.

Apart from changes in wax scent amounts with increasing worker number, the wax scent also changed with regard to the composition of the relative compound amounts between the social phase and competition phase. In the four colonies included in our analyses, the lipid pattern of nest wax changed over time. Interestingly, the wax profiles of all colonies changed in a similar manner irrespective of colony identity. Differences in wax scent patterns between social-phase and competition-phase colonies were mainly caused by alterations in the relative amounts of odd-numbered alkanes, various alkenes and alkadienes, and oxygenated cuticular lipids, i.e. wax esters, the ethyl ester ethyl hexadecanoate, aldehydes and the ketone 2-nonacosanone. Interestingly, we also found a slight increase in the relative amounts of pentacosane (C25) in competition-phase wax. This alkane dominates the cuticle-surface profile of breeding queens and is considered queen-caste specific ([22], own data, see the electronic supplementary material, appendix B). It has been shown to cause secondary oocyte resorption in queenless colonies [22]. However, our bioassays indicate that the presence of C25 in competition phase wax is not enough to prevent competitive behaviour and increased fertility in callow worker groups.

Hydrocarbons are known to play an important role in the communication systems of many social insects (reviewed in [57]). In bumblebees, hydrocarbons are involved in fertility and task signalling [22–24,48], in egg marking [49], in foraging scent-marking [58,59] and in nest recognition [46,51,52]. Several studies on bumblebees have indicated that, in addition to hydrocarbons, oxygenated compounds such as ethyl esters and wax esters that are produced in various bumblebee glands [60,61] can have behaviour-modulating effects. The wax esters that have been found in higher relative amounts in competition-phase wax extracts signal fertility in various bumblebee species [24,25]. Other esters signal worker sterility thereby preventing attacks on non-competing nest-mates [61].

### 4.2 Origin of changes in wax scent profiles

Wax scent represents the average cuticle-surface profile of all colony members [46] as every colony member contributes cuticular compounds to the wax profile when producing wax scales and leaving footprints on the wax surface [50,51,62]. Thus, changes in wax scent patterns during colony development should be caused either by changes in the individual cuticle-surface profiles of all colony members or by the composition of the workforce (subcastes with differing scent profiles). Studies on ants have shown that the average composition of cuticle profiles of one colony can change considerably within half a month [63–66]. Seasonal changes and differences in diet seem to be the driving force for this temporal diversity [65,67]. However, an influence of these parameters can be excluded in our study as the bumblebee colonies have been kept under constant laboratory conditions.

As the wax scent changed in a similar manner in all bumblebee colonies observed, the dynamics in wax scent were more likely determined by characteristic changes in the composition of the workforce with colony development, e.g. the higher number of fertile workers in competition-phase colonies. With regard to the subset of wax lipids that was affected by increasing colony age, many of the compounds that increased in their relative amounts in all colonies were noticeably associated with fertility in both queens and workers [24,25].

Our comparison of the Bray–Curtis similarities between wax profiles and surface profiles of adult bumblebees showed that substance patterns from competition-phase wax indeed became more similar to the patterns of cuticle-surface profiles of workers.

The increased similarity of wax profiles in competition-phase colonies to cuticle-surface profiles of fertile workers might be explained by a higher total number of fertile workers in a later stage of colony development. In expectation of a similar wax production rate in all workers (remains to be tested in future investigations) and on the assumption of a continuum between nest wax and the cuticle surface of all colony members [46], fertile workers are likely to contribute higher amounts of cuticular lipids to the joint nest wax scent than sterile workers, as they have a 2.5 times higher concentration of cuticle-surface compounds than sterile nest-mates (concentration of cuticular lipids (mg) on fertile workers: 0.2, sterile workers: 0.08, breeding queens: 1.4 [24]). The increasing number of fertile workers during colony development could thus lead to an increase of the proportion of compounds that are prevalent in the cuticle lipid pattern of fertile workers.

So far, our analyses of the wax chemistry indicate that wax compound amounts and profile patterns mirror the actual social condition of bumblebee colonies in representing colony age, the total size of the workforce and the presence of a high number of fertile workers. Thus, wax cues can provide information that workers need to time their reproduction [28,36–38]. Further alterations in wax scent might be caused by a diminished concentration of pheromone substances associated with the queen. In honeybees, wax has been suggested to be an important medium and sink for the queen retinue pheromone in honeybees [68]. In larger colonies, these substances might be diluted by an increased overall colony scent [69]. Additionally, substances associated with the rearing of queen larvae could also influence the competition-phase wax scent. Several studies of the conflict over male production have suggested that the presence of queen larvae at a later stage of colony development influences the onset of competition over reproduction [16,36,38–41] and that a shift in queen signalling might be responsible for the changing fate of diploid larvae [38,40,42]. At the moment, we are testing whether wax also contains chemical compounds that are associated with the queen or with queen larvae. Such compounds might be present in wax extracts at low concentrations beyond the detection limit of the present chemical analyses. Furthermore, it will be interesting to test whether queen and workers contribute equally to the wax scent, or if for example fertile female bumblebees produce more wax scales which might explain the increased similarity of competition-phase wax profiles to the profiles of fertile workers.

### 4.3 Effect of wax on worker behaviour and physiology

In our behavioural assays, wax did indeed have an impact on worker reproduction in bumblebees. Queen-right groups of newly emerged workers challenged with a competition-phase wax environment started to show aggressive interactions. Such aggressive interactions are commonly observed in queenless worker groups and during the first few days after the onset of competition in queen-right colonies [15,27]. We have been able to show that the competition-phase wax environment elicited a similar amount of aggression in queen-right worker groups. In the presence of a fertile queen, a group of 10 callow workers would normally refrain from biting and stinging attempts [37], as we have also observed in all other treatments, i.e. groups with social-phase wax, unimpaired social-phase colonies and without additional information from the nest environment.

Interestingly, we found no changes in the queens’ behaviour between the different treatments. There were no signs of agitation or increased aggressiveness in the queens that were grouped with workers that competed for reproduction in the competition-phase wax environment. Several studies suggested that during the competition phase the workers are the major factor inhibiting the reproduction of their nest-mates [9,15,19]. As worker reproduction goes along with violent, even fatal, interactions between members of the colony, including egg-eating, fights within the nest and occasionally the death of the queen [17], it might be adaptive for queens to avoid aggressive interactions with workers.

Apart from changes in the behaviour of workers, the presence of competition-phase wax also enhanced the development of worker ovaries. Despite an identical group size and the presence of a breeding queen, the ovaries of newly emerged workers that encounter wax from a competition-phase colony develop faster than those in any other treatment examined. The ovarian development of callow workers in groups with competition-phase wax was comparable with that of queenless groups of callow workers in which about 40% of workers develop ovaries containing ripe eggs within 6–7 days after emergence [10,11,27]. However, in queen-right social-phase colonies, the development of worker ovaries is slower and the utmost proportion of potential egg layers (40%) is reached only among 10-day-old workers [11]. In our group experiments, the delaying effect of the queen’s presence on the development of worker ovaries is maintained in groups that encounter social-phase wax, social-phase colonies or an empty nest environment. However, it was entirely lost in groups in which competition-phase wax was present. This finding agrees with observations of van Doorn & Heringa [15], who have shown that some queen-right workers that have emerged shortly after the onset of competition phase ‘escape’ the inhibiting power of the queen and the elite group and can become the youngest egg layers of the colony as they develop their ovaries faster within 6 days, compared with queen-right workers that need 10 days to gain ripe ovaries during the social phase of colony development.

Thus, workers seem to be able to overcome the ovarian inhibition by the queen depending on the social context. We still need to test which compounds within wax lipid profiles, their quantitative composition and/or concentration are indeed responsible for alterations in the reproductive behaviour and physiology of workers that we have found in our group experiments with newly emerged workers. As profile and concentration changes might occur gradually, an examination of the point at which changes in patterns or compound concentrations are sufficient to trigger aggression and enhance ovarian development will be of interest. A threshold might exist that, once reached, elicits a standard response in fertile workers, i.e. they stop cooperating in favour of own reproduction. Although further experiments will be needed to establish that changes in wax scent are responsible for the transition from the social phase to competition phase, our findings from queen-right worker groups clearly show that the presence of competition-phase wax considerably influences worker behaviour and the physiology of newly emerged workers, as would be expected in the competition phase only.

In summary, we could show that the joint signature scent of all colony members present in nest wax not only functions as a colony-specific cue indicating nest identity [46]. Nest wax scent also mirrors the social condition of the colony, i.e. changes in the size of the workforce and the presence of fertile workers. This adds a new dimension to the role of nest wax in bumblebee communication and emphasizes the importance of cues from the nest environment in the organization and maintenance of social insect colonies. Our behavioural experiments clearly revealed that nest wax can influence reproductive behaviour and ovarian development in bumblebee workers. Most probably, the changes in the composition of lipids and/or differences in the wax scent concentration stimulated these alterations in worker reproductive behaviour and physiology. In providing knowledge of the size and composition of the workforce, nest wax scent could help workers to time reproduction without diminishing the overall colony performance and gyne production. In our experiments, the onset of worker reproduction was triggered by the presence of competition-phase wax, independently of the actual group size and status of their queen. When exposed to contradictory information of a fertile and active queen versus wax that indicates a competition-phase colony, the information gained from wax outweighed the queen’s influence. Our results shed new light on the proximate mechanisms underlying the characteristic shift from cooperation to selfish behaviour in bumblebee workers and support the view of recent studies on worker reproduction in bumblebees indicating that *B. terrestris* workers are able to auto-regulate their reproduction, despite queen control at a certain point of colony development [28,38,40].

## Supplementary Material

Appendix A

## Supplementary Material

Appendix B

## Supplementary Material

Data Rottler et al Proc R Soc

## Data accessibility

The datasets supporting this article have been uploaded as part of the electronic supplementary material. They have also been deposited in the Dryad Digital Repository: http://dx.doi.org/10.5061/dryad.kd1r2.
