# Supplementary material for: Nest wax triggers worker reproduction in the bumblebee Bombus terrestris
Source: R Soc Open Sci. 2016 Jan 6;3(1):150599. doi: 10.1098/rsos.150599 (PMC4736944; doi:10.1098/rsos.150599)
Supplement: Appendix A [file rsos150599supp1.pdf]

**Appendix A.** Effect of colony identity, colony age and wax type on the relative amounts of 76 compounds extracted from bumblebee wax samples (GLMM statistics). Compounds are sorted by substance class (italics) and the numbers given with the compound names encode peak identity (main text figure 1). $P$-values marked bold indicate significant effects.

| Compounds | Corrected model ($df_1$=7, $df_2$=72) | Colony identity ($df_1$=3, $df_2$=72) | Colony age ($df_1$=3, $df_2$=72) | Wax type ($df_1$=1, $df_2$=72) |
|---|---|---|---|---|
| *Alkanes* | | | | |
| **Henicosane (2)** | $F$=19.903, **$P$<0.001** | $F$=0.289, $P$=0.834 | $F$=45.415, **$P$<0.001** | $F$=2.213, $P$=0.141 |
| **Docosane (3)** | $F$=25.208, **$P$<0.001** | $F$=27.812, **$P$<0.001** | $F$=30.448, **$P$<0.001** | $F$=1.676, $P$=0.200 |
| **Tricosane (5)** | $F$=13.824, **$P$<0.001** | $F$=1.478, $P$=0.228 | $F$=30.768, **$P$<0.001** | $F$=0.027, $P$=0.871 |
| Tetracosane (6) | $F$=3.219, **$P$=0.005** | $F$=0.715, $P$=0.546 | $F$=6.725, **$P$<0.001** | $F$=0.209, $P$=0.649 |
| Pentacosane (11) | $F$=2.466, **$P$=0.025** | $F$=1.261, $P$=0.294 | $F$=4.395, **$P$=0.007** | $F$=0.297, $P$=0.587 |
| **Hexacosane (14)** | $F$=11.258, **$P$<0.001** | $F$=0.000, $P$=1.000 | $F$=26.076, **$P$<0.001** | $F$=0.579, $P$=0.449 |
| **Heptacosane (21)** | $F$=11.140, **$P$<0.001** | $F$=0.923, $P$=0.434 | $F$=24.220, **$P$<0.001** | $F$=2.553, $P$=0.114 |
| Octacosane (26) | $F$=4.628, **$P$<0.001** | $F$=0.000, $P$=1.000 | $F$=10.045, **$P$<0.001** | $F$=2.263, $P$=0.137 |
| Nonacosane (33) | $F$=8.013, **$P$<0.001** | $F$=0.372, $P$=0.773 | $F$=18.324, **$P$<0.001** | $F$=0.000, $P$=1.000 |
| Triacontane (38) | $F$=2.250, **$P$=0.040** | $F$=0.057, $P$=0.982 | $F$=3.364, **$P$=0.023** | $F$=5.492, **$P$=0.022** |
| Hentriacontane (47) | $F$=1.638, $P$=0.139 | $F$=0.477, $P$=0.699 | $F$=1.851, $P$=0.146 | $F$=4.479, **$P$=0.038** |
| Tritriacontane (56) | $F$=9.058, **$P$<0.001** | $F$=0.427, $P$=0.735 | $F$=20.624, **$P$<0.001** | $F$=0.254, $P$=0.616 |
| *Alkenes* | | | | |
| (Z)-9-Tricosene (4) | $F$=0.542, $P$=0.800 | $F$=0.046, $P$=0.987 | $F$=0.997, $P$=0.399 | $F$=0.666, $P$=0.417 |
| (Z)-10-Pentacosene (7) | $F$=4.328, **$P$<0.001** | $F$=0.000, $P$=1.000 | $F$=8.978, **$P$<0.001** | $F$=3.361, $P$=0.071 |
| (Z)-9-Pentacosene (8) | $F$=4.881, **$P$<0.001** | $F$=6.423, **$P$=0.001** | $F$=4.253, **$P$=0.008** | $F$=2.140, $P$=0.148 |
| (Z)-8-Pentacosene (9) | $F$=0.043, $P$=1.000 | $F$=0.016, $P$=0.997 | $F$=0.074, $P$=0.974 | $F$=0.035, $P$=0.851 |
| (Z)-7-Pentacosene (10) | $F$=1.516, $P$=0.176 | $F$=0.000, $P$=1.000 | $F$=2.778, **$P$=0.047** | $F$=2.281, $P$=0.135 |
| (Z)-11- and (Z)-10-Heptacosene (16) | $F$=4.080, **$P$=0.001** | $F$=0.200, $P$=0.896 | $F$=9.158, **$P$<0.001** | $F$=0.488, $P$=0.487 |
| (Z)-9-Heptacosene (17) | $F$=5.573, **$P$<0.001** | $F$=0.511, $P$=0.676 | $F$=12.469, **$P$<0.001** | $F$=0.068, $P$=0.795 |
| (Z)-8-Heptacosene (18) | $F$=0.873, $P$=0.532 | $F$=0.564, $P$=0.640 | $F$=0.729, $P$=0.538 | $F$=2.229, $P$=0.140 |
| **(Z)-7-Heptacosene (19)** | $F$=12.481, **$P$<0.001** | $F$=13.066, **$P$<0.001** | $F$=15.907, **$P$<0.001** | $F$=0.447, $P$=0.506 |
| Octacosene* (25) | $F$=1.792, $P$=0.102 | $F$=0.052, $P$=0.984 | $F$=3.277, **$P$=0.026** | $F$=2.554, $P$=0.114 |
| (Z)-11-Nonacosene (30) | $F$=8.410, **$P$<0.001** | $F$=0.198, $P$=0.898 | $F$=19.323, **$P$<0.001** | $F$=0.307, $P$=0.581 |
| (Z)-9-Nonacosene (31) | $F$=5.788, **$P$<0.001** | $F$=0.662, $P$=0.578 | $F$=12.828, **$P$<0.001** | $F$=0.047, $P$=0.828 |
| (Z)-7-Nonacosene (32) | $F$=2.855, **$P$=0.011** | $F$=0.004, $P$=1.000 | $F$=6.510, **$P$=0.001** | $F$=0.446, $P$=0.506 |
| Triacontene* (35) | $F$=5.350, **$P$<0.001** | $F$=0.228, $P$=0.877 | $F$=10.637, **$P$<0.001** | $F$=4.853, **$P$=0.031** |
| Triacontene* (36) | $F$=5.459, **$P$<0.001** | $F$=0.213, $P$=0.887 | $F$=12.079, **$P$<0.001** | $F$=1.338, $P$=0.251 |
| Triacontene* (37) | $F$=3.566, **$P$=0.002** | $F$=0.654, $P$=0.583 | $F$=7.230, **$P$<0.001** | $F$=1.312, $P$=0.256 |
| Hentriacontene* (42) | $F$=7.265, **$P$<0.001** | $F$=0.000, $P$=1.000 | $F$=16.791, **$P$<0.001** | $F$=0.483, $P$=0.490 |
| Hentriacontene* (43) | $F$=5.745, **$P$<0.001** | $F$=9.603, **$P$<0.001** | $F$=3.772, **$P$=0.014** | $F$=0.091, $P$=0.764 |
| **Hentriacontene* (44)** | $F$=19.529, **$P$<0.001** | $F$=0.203, $P$=0.894 | $F$=45.353, **$P$<0.001** | $F$=0.033, $P$=0.856 |
| **Hentriacontene* (45)** | $F$=38.287, **$P$<0.001** | $F$=1.386, $P$=0.254 | $F$=87.947, **$P$<0.001** | $F$=0.009, $P$=0.926 |
| Tritriacontene* (54) | $F$=1.502, $P$=0.181 | $F$=0.418, $P$=0.741 | $F$=3.066, **$P$=0.033** | $F$=0.060, $P$=0.807 |
| **Tritriacontene* (55)** | $F$=25.550, **$P$<0.001** | $F$=4.423, **$P$=0.007** | $F$=54.344, **$P$<0.001** | $F$=2.547, $P$=0.115 |
| Pentatriacontene* (63) | $F$=6.051, **$P$<0.001** | $F$=3.202, **$P$=0.028** | $F$=10.558, **$P$<0.001** | $F$=1.075, $P$=0.303 |
| *Alkadienes* | | | | |
| Nonacosadiene* (28) | $F$=1.105, $P$=0.369 | $F$=0.032, $P$=0.992 | $F$=2.544, $P$=0.063 | $F$=0.008, $P$=0.931 |
| Nonacosadiene* (29) | $F$=2.944, **$P$=0.009** | $F$=0.002, $P$=1.000 | $F$=5.690, **$P$<0.001** | $F$=3.532, $P$=0.064 |
| Triacontadiene* (34) | $F$=3.478, **$P$=0.003** | $F$=0.835, $P$=0.479 | $F$=7.120, **$P$<0.001** | $F$=0.480, $P$=0.491 |
| Hentriacontadiene* (40) | $F$=1.388, $P$=0.224 | $F$=0.014, $P$=0.998 | $F$=2.386, $P$=0.076 | $F$=2.513, $P$=0.117 |
| Hentriacontadiene* (41) | $F$=9.044, **$P$<0.001** | $F$=0.003, $P$=1.000 | $F$=21.075, **$P$<0.001** | $F$=0.070, $P$=0.791 |
| Tritriacontadiene* (51) | $F$=1.594, $P$=0.151 | $F$=0.094, $P$=0.963 | $F$=2.421, $P$=0.073 | $F$=3.614, $P$=0.061 |
| Tritriacontadiene* (52) | $F$=9.942, **$P$<0.001** | $F$=0.001, $P$=1.000 | $F$=23.196, **$P$<0.001** | $F$=0.006, $P$=0.939 |

Continued on next page.

| Compounds | Corrected model ($df_1$=7, $df_2$=72) | Colony identity ($df_1$=3, $df_2$=72) | Colony age ($df_1$=3, $df_2$=72) | Wax type ($df_1$=1, $df_2$=72) |
|---|---|---|---|---|
| **Tritriacontadiene* (53)** | *F*=10.255, ***P*<0.001** | *F*=0.336, *P*=0.800 | *F*=23.591, ***P*<0.001** | *F*=0.003, *P*=0.955 |
| Pentatriacontadiene* (61) | *F*=5.142, ***P*<0.001** | *F*=7.921, ***P*<0.001** | *F*=2.590, *P*=0.059 | *F*=4.456, *P*=0.038 |
| *Methylated alkanes* | | | | |
| 11-Methylpentacosane, 9-Methylpentacosane (12) | *F*=0.833, *P*=0.563 | *F*=0.049, *P*=0.986 | *F*=1.409, *P*=0.247 | *F*=1.460, *P*=0.231 |
| 5-Methylpentacosane (13) | *F*=2.339, ***P*=0.033** | *F*=0.639, *P*=0.592 | *F*=4.810, ***P*=0.004** | *F*=0.024, *P*=0.878 |
| 13-Methylheptacosane (22) | *F*=2.483, ***P*=0.024** | *F*=0.269, *P*=0.848 | *F*=5.182, ***P*=0.003** | *F*=1.030, *P*=0.313 |
| 11-Methylheptacosane (23) | *F*=1.372, *P*=0.230 | *F*=0.327, *P*=0.806 | *F*=2.826, ***P*=0.045** | *F*=0.147, *P*=0.702 |
| 3-Methylheptacosane (24) | *F*=0.893, *P*=0.516 | *F*=0.078, *P*=0.972 | *F*=1.177, *P*=0.325 | *F*=2.487, *P*=0.119 |
| *Ethylesters* | | | | |
| Ethyl hexadecanoate (1) | *F*=4.674, ***P*<0.001** | *F*=0.075, *P*=0.973 | *F*=9.513, ***P*<0.001** | *F*=3.766, *P*=0.056 |
| Ethyl octacosanoate (50) | *F*=0.603, *P*=0.751 | *F*=0.120, *P*=0.948 | *F*=0.594, *P*=0.621 | *F*=2.078, *P*=0.154 |
| Ethyl triacontanoate (60) | *F*=4.595, ***P*<0.001** | *F*=0.501, *P*=0.683 | *F*=9.490, ***P*<0.001** | *F*=2.195, *P*=0.143 |
| *Wax esters* | | | | |
| **Hexadecyl tetradecanoate (49)** | *F*=12.686, ***P*<0.001** | *F*=0.112, *P*=0.953 | *F*=25.786, ***P*<0.001** | *F*=11.108, ***P*=0.001** |
| Icosyl hexadecanoate (66) | *F*=2.026, *P*=0.063 | *F*=0.718, *P*=0.544 | *F*=3.515, ***P*=0.019** | *F*=1.486, *P*=0.227 |
| **Icosyl octadecenoate* (67)** | *F*=10.527, ***P*<0.001** | *F*=14.328, ***P*<0.001** | *F*=10.221, ***P*<0.001** | *F*=0.041, *P*=0.840 |
| Docosyl hexadecanoate, Icosyl octadecanoate (68) | *F*=1.259, *P*=0.283 | *F*=0.176, *P*=0.912 | *F*=1.878, *P*=0.141 | *F*=2.652, *P*=0.108 |
| **Docosyl octadecenoate* (69)** | *F*=10.330, ***P*<0.001** | *F*=17.753, ***P*<0.001** | *F*=6.188, ***P*=0.001** | *F*=0.488, *P*=0.487 |
| Tetracosyl hexadecanoate, Docosyl octadecanoate (70) | *F*=0.614, *P*=0.742 | *F*=0.002, *P*=1.000 | *F*=1.026, *P*=0.386 | *F*=1.216, *P*=0.274 |
| Tetracosyl octadecenoate* (71) | *F*=3.137, ***P*=0.006** | *F*=0.407, *P*=0.749 | *F*=5.722, ***P*=0.001** | *F*=3.571, *P*=0.063 |
| Hexacosyl hexadecanoate, Tetracosyl octadecanoate (72) | *F*=0.274, *P*=0.962 | *F*=0.025, *P*=0.995 | *F*=0.468, *P*=0.705 | *F*=0.434, *P*=0.512 |
| **Hexacosyl octadecenoate* (73)** | *F*=12.692, ***P*<0.001** | *F*=0.190, *P*=0.903 | *F*=29.141, ***P*<0.001** | *F*=0.847, *P*=0.360 |
| Octacosyl hexadecanoate, Hexacosyl octadecanoate (74) | *F*=1.612, *P*=0.146 | *F*=0.007, *P*=0.999 | *F*=3.367, ***P*=0.023** | *F*=1.166, *P*=0.284 |
| Octacosyl octadecenoate* (75) | *F*=4.219, ***P*=0.001** | *F*=0.364, *P*=0.779 | *F*=9.464, ***P*<0.001** | *F*=0.047, *P*=0.828 |
| Triacontyl octadecenoate* (76) | *F*=0.105, *P*=0.998 | *F*=0.070, *P*=0.976 | *F*=0.173, *P*=0.914 | *F*=0.002, *P*=0.962 |
| *Aldehydes* | | | | |
| Triacontanal (58) | *F*=2.178, ***P*=0.046** | *F*=2.197, *P*=0.096 | *F*=1.907, *P*=0.136 | *F*=2.935, *P*=0.091 |
| Dotriacontanal (65) | *F*=3.939, ***P*=0.001** | *F*=0.004, *P*=1.000 | *F*=8.408, ***P*<0.001** | *F*=2.339, *P*=0.131 |
| *Ketones* | | | | |
| 2-Nonacosanone (48) | *F*=3.085, ***P*=0.007** | *F*=0.016, *P*=0.997 | *F*=5.580, ***P*=0.002** | *F*=4.807, ***P*=0.032** |
| **2-Triacontanone (59)** | *F*=14.239, ***P*<0.001** | *F*=0.550, *P*=0.650 | *F*=20.564, ***P*<0.001** | *F*=36.330, ***P*<0.001** |
| *Acetates* | | | | |
| Tetracosyl acetate (27) | *F*=0.442, *P*=0.872 | *F*=0.000, *P*=1.000 | *F*=0.645, *P*=0.589 | *F*=1.159, *P*=0.285 |
| Hexacosyl acetate (39) | *F*=1.866, *P*=0.088 | *F*=0.254, *P*=0.858 | *F*=4.020, ***P*=0.011** | *F*=0.240, *P*=0.626 |
| *Unidentified* | | | | |
| Unidentified (15) | *F*=1.904, *P*=0.081 | *F*=0.510, *P*=0.676 | *F*=2.601, *P*=0.059 | *F*=3.996, ***P*=0.049** |
| Unidentified (20) | *F*=1.313, *P*=0.257 | *F*=0.422, *P*=0.737 | *F*=2.601, *P*=0.059 | *F*=0.121, *P*=0.729 |
| **Unidentified (46)** | *F*=27.227, ***P*<0.001** | *F*=35.382, ***P*<0.001** | *F*=28.030, ***P*<0.001** | *F*=0.355, *P*=0.553 |
| Unidentified (57) | *F*=3.823, ***P*=0.001** | *F*=0.427, *P*=0.734 | *F*=7.465, ***P*<0.001** | *F*=3.088, *P*=0.083 |
| Unidentified (62) | *F*=7.440, ***P*<0.001** | *F*=14.028, ***P*<0.001** | *F*=1.787, *P*=0.157 | *F*=4.636, ***P*=0.035** |
| Unidentified (64) | *F*=3.953, ***P*=0.001** | *F*=0.832, *P*=0.481 | *F*=8.218, ***P*<0.001** | *F*=0.522, *P*=0.472 |

*asterik*: unknown double bond position
