# Supplementary material for: Nest wax triggers worker reproduction in the bumblebee Bombus terrestris
Source: R Soc Open Sci. 2016 Jan 6;3(1):150599. doi: 10.1098/rsos.150599 (PMC4736944; doi:10.1098/rsos.150599)
Supplement: Appendix B [file rsos150599supp2.pdf]

Appendix B. Comparison of the relative amounts of 76 compounds examined in wax extracts and bumblebee extracts (median, 25th and 75th percentile; Kruskal-Wallis with *post-hoc* Dunn's test statistics). Compounds are sorted by substance class (italics) and the numbers given with the compound names encode peak identity (main text figure 1).

Compounds	Sp wax (n = 40)	Cp wax (n = 40)	Sterile worker (n = 31)	Fertile worker (n = 50)	Queen (n = 9)	H_4	p
Alkanes							
Henicosane (2)	0.33 (0.31, 0.41)[a]	0.46 (0.40, 0.55)[b]	0.79 (0.58, 1.11)[c]	0.53 (0.44, 0.70)[bc]	0.43 (0.37, 0.51)[ab]	73.79	<0.001
Docosane (3)	0.15 (0.14, 0.16)[a]	0.18 (0.16, 0.19)[a]	0.21 (0.20, 0.26)[b]	0.23 (0.21, 0.27)[b]	0.26 (0.21, 0.29)[b]	102.89	<0.001
Tricosane (5)	10.40 (9.96, 11.03)[a]	12.11 (10.44, 12.41)[a]	13.07 (11.56, 14.96)[b]	15.21 (14.19, 16.95)[b]	15.94 (13.65, 17.56)[b]	98.67	<0.001
Tetracosane (6)	0.47 (0.45, 0.49)[a]	0.48 (0.44, 0.49)[a]	0.35 (0.27, 0.43)[b]	0.55 (0.48, 0.63)[c]	0.67 (0.64, 0.70)[c]	72.83	<0.001
Pentacosane (11)	15.95 (15.39, 16.40)[a]	16.30 (15.29, 16.84)[a]	12.28 (8.85, 14.47)[b]	18.38 (14.91, 20.42)[ac]	20.44 (20.20, 21.80)[c]	67.30	<0.001
Hexacosane (14)	0.75 (0.74, 0.77)[a]	0.71 (0.69, 0.73)[b]	0.63 (0.52, 0.71)[c]	0.67 (0.60, 0.72)[bc]	0.80 (0.77, 0.83)[a]	69.34	<0.001
Heptacosane (21)	15.85 (15.24, 16.26)[a]	14.92 (14.52, 15.37)[b]	14.12 (12.58, 15.61)[bc]	12.65 (11.57, 14.42)[c]	14.86 (13.46, 16.14)[ab]	63.94	<0.001
Octacosane (26)	0.37 (0.35, 0.39)[a]	0.34 (0.32, 0.35)[b]	0.37 (0.30, 0.43)[ab]	0.25 (0.21, 0.30)[c]	0.35 (0.31, 0.40)[ab]	78.71	<0.001
Nonacosane (33)	6.88 (6.59, 7.50)[a]	6.24 (5.88, 6.75)[b]	6.56 (5.28, 8.50)[ab]	4.12 (3.10, 4.97)[c]	4.93 (4.42, 6.23)[bc]	87.56	<0.001
Triacontane (38)	0.08 (0.07, 0.10)[a]	0.07 (0.06, 0.08)[a]	0.02 (0.01, 0.03)[b]	0.02 (0.01, 0.03)[b]	0.09 (0.07, 0.10)[a]	86.41	<0.001
Hentriacontane (47)	0.48 (0.43, 0.55)[a]	0.45 (0.36, 0.52)[a]	0.23 (0.16, 0.29)[b]	0.18 (0.13, 0.23)[b]	0.09 (0.08, 0.13)[b]	109.56	<0.001
Tritriacontane (56)	0.07 (0.06, 0.08)[a]	0.11 (0.09, 0.12)[b]	0.07 (0.06, 0.10)[a]	0.07 (0.06, 0.09)[ab]	0.08 (0.07, 0.11)[ab]	34.52	<0.001
Alkenes							
(Z)-9-Tricosene (4)	0.00 (0.00, 0.00)[a]	0.00 (0.00, 0.00)[a]	0.15 (0.10, 0.18)[b]	0.25 (0.19, 0.28)[b]	0.16 (0.12, 0.22)[b]	134.93	<0.001
(Z)-10-Pentacosene (7)	0.03 (0.02, 0.03)[a]	0.03 (0.03, 0.04)[b]	0.05 (0.04, 0.07)[c]	0.05 (0.04, 0.07)[c]	0.04 (0.04, 0.06)[bc]	75.83	<0.001
(Z)-9-Pentacosene (8)	0.16 (0.14, 0.17)[a]	0.16 (0.14, 0.17)[a]	0.09 (0.07, 0.13)[b]	0.16 (0.12, 0.25)[a]	0.16 (0.11, 0.21)[a]	29.64	<0.001
(Z)-8-Pentacosene (9)	0.02 (0.02, 0.03)[a]	0.03 (0.02, 0.03)[ab]	0.03 (0.02, 0.03)[bc]	0.04 (0.03, 0.04)[c]	0.04 (0.03, 0.04)[c]	45.45	<0.001
(Z)-7-Pentacosene (10)	0.05 (0.05, 0.06)[ab]	0.05 (0.05, 0.05)[a]	0.04 (0.03, 0.05)[a]	0.06 (0.05, 0.08)[b]	0.05 (0.05,0.06)[ab]	21.92	<0.001
(Z)-11- and (Z)-10-Heptacosene (16)	0.06 (0.05, 0.07)[a]	0.07 (0.06, 0.09)[a]	0.18 (0.13, 0.40)[b]	0.11 (0.09, 0.14)[b]	0.10 (0.07, 0.12)[ab]	80.31	<0.001
(Z)-9-Heptacosene (17)	0.47 (0.44, 0.49)[ab]	0.48 (0.43, 0.52)[a]	0.38 (0.31, 0.50)[b]	0.49 (0.40, 0.72)[a]	0.35 (0.29, 0.54)[ab]	14.50	0.006
(Z)-8-Heptacosene (18)	0.09 (0.07, 0.09)[a]	0.10 (0.09, 0.11)[a]	0.17 (0.15, 0.21)[b]	0.10 (0.08, 0.13)[a]	0.11 (0.08, 0.12)[a]	75.17	<0.001
(Z)-7-Heptacosene (19)	0.41 (0.37, 0.46)[ab]	0.41 (0.37, 0.46)[ab]	0.35 (0.28, 0.45)[a]	0.48 (0.41, 0.71)[b]	0.29 (0.27, 0.36)[a]	33.23	<0.001
Octacosene* (25)	0.23 (0.22, 0.34)[a]	0.22 (0.21, 0.25)[a]	0.22 (0.19, 0.28)[a]	0.18 (0.16, 0.22)[b]	0.15 (0.12, 0.19)[b]	52.48	<0.001
(Z)-11-Nonacosene (30)	0.79 (0.73, 0.87)[a]	0.92 (0.84, 1.07)[a]	2.24 (1.54, 3.36)[b]	0.88 (0.77, 1.10)[a]	0.78 (0.59, 0.88)[a]	81.87	<0.001
(Z)-9-Nonacosene (31)	5.17 (4.95, 5.29)[a]	4.94 (4.57, 5.37)[ab]	5.33 (4.21, 7.46)[a]	4.42 (3.44, 5.52)[b]	3.28 (2.15, 3.97)[c]	31.27	<0.001
(Z)-7-Nonacosene (32)	0.84 (0.69, 0.95)[ab]	0.79 (0.73, 0.89)[a]	1.49 (1.03, 2.03)[b]	0.64 (0.53, 0.85)[c]	0.75 (0.66, 1.00)[abc]	36.20	<0.001

Continued on next page.

Compounds	Sp wax (*n* = 40)	Cp wax (*n* = 40)	Sterile worker (*n* = 31)	Fertile worker (*n* = 50)	Queen (*n* = 9)	H_4	*p*
Triacontene* (35)	0.04 (0.03, 0.05)[a]	0.05 (0.04, 0.06)[a]	0.13 (0.08, 0.17)[b]	0.06 (0.04, 0.08)[a]	0.06 (0.05, 0.06)[ab]	70.74	<0.001
Triacontene* (36)	0.07 (0.06, 0.08)[a]	0.08 (0.07, 0.10)[b]	0.19 (0.18, 0.28)[c]	0.08 (0.06, 0.12)[ab]	0.07 (0.06, 0.08)[ab]	76.87	<0.001
Triacontene* (37)	0.24 (0.23, 0.26)[a]	0.21 (0.19, 0.23)[b]	0.21 (0.18, 0.27)[ab]	0.20 (0.16, 0.22)[b]	0.16 (0.14, 0.21)[b]	35.51	<0.001
Hentriacontene* (42)	0.64 (0.61, 0.66)[a]	0.72 (0.68, 0.75)[a]	1.20 (0.92, 1.52)[b]	1.27 (0.93, 1.43)[b]	0.74 (0.52, 0.79)[a]	100.75	<0.001
Hentriacontene* (43)	0.83 (0.80, 0.88)[a]	0.81 (0.75, 0.88)[a]	1,26 (0.98, 1.55)[b]	1.22 (0.96, 1.51)[b]	0.77 (0.69, 0.97)[a]	72.08	<0.001
Hentriacontene* (44)	1.72 (1.60, 1.82)[a]	1.96 (1.90, 2.37)[ab]	7.03 (4.66, 8.38)[a]	2.87 (2.36, 3.62)[ab]	2.62 (2.14, 3.01)[b]	113.31	<0.001
Hentriacontene* (45)	5.51 (5.11, 5.72)[a]	4.67 (4.21, 4.95)[b]	5.44 (4.87, 6.65)[a]	5.05 (4.43, 5.76)[ac]	4.04 (3.48, 5.19)[bc]	37.48	<0.001
Tritriacontene* (54)	0.41 (0.35, 0.45)[a]	0.47 (0.41, 0.55)[a]	1.17 (0.87, 1.62)[b]	1.12 (0.72, 1.62)[b]	1.00 (0.77, 1.28)[b]	91.76	<0.001
Tritriacontene* (55)	0.32 (0.25, 0.37)[ab]	0.23 (0.17, 0.28)[c]	0.53 (0.32, 0.94)[a]	0.28 (0.18, 0.38)[bc]	0.42 (0.31, 0.46)[ab]	47.32	<0.001
Pentatriacontene* (63)	0.08 (0.07, 0.10)[ab]	0.11 (0.08, 0.13)[a]	0.06 (0.04, 0.09)[b]	0.07 (0.04, 0.10)[b]	0.11 (0.08, 0.16)[a]	37.34	<0.001
Alkadienes							
Nonacosadiene* (28)	0.04 (0.04, 0.05)[a]	0.05 (0.05, 0.06)[a]	0.06 (0.03, 0.15)[a]	0.06 (0.03, 0.09)[a]	0.05 (0.03, 0.06)[a]	9.62	0.047
Nonacosadiene* (29)	0.15 (0.12, 0.16)[a]	0.16 (0.15, 0.17)[ab]	0.20 (0.13, 0.28)[b]	0.16 (0.12, 0.21)[ab]	0.13 (0.10, 0.15)[a]	17.45	0.002
Triacontadiene* (34)	0.06 (0.05, 0.08)[a]	0.08 (0.07, 0.09)[b]	0.07 (0.05, 0.10)[ab]	0.04 (0.03, 0.07)[b]	0.04 (0.03, 0.05)[b]	35.33	<0.001
Hentriacontadiene* (40)	0.21 (0.14, 0.28)[a]	0.22 (0.17, 0.28)[a]	0.55 (0.35, 0.90)[b]	0.33 (0.26, 0.42)[b]	0.21 (0.16, 0.22)[a]	67.45	<0.001
Hentriacontadiene* (41)	0.21 (0.19, 0.24)[a]	0.29 (0.26, 0.32)[b]	0.75 (0.55, 0.93)[c]	0.54 (0.46, 0.70)[c]	0.45 (0.40, 0.53)[bc]	125.15	<0.001
Tritriacontadiene* (51)	0.31 (0.16, 0.42)[ab]	0.18 (0.13, 0.27)[a]	0.37 (0.24, 0.54)[b]	0.46 (0.22, 0.62)[b]	0.27 (0.16, 0.35)[ab]	21.73	<0.001
Tritriacontadiene* (52)	0.36 (0.32, 0.42)[a]	0.58 (0.51, 0.69)[ab]	1.25 (0.98, 1.51)[c]	1.54 (1.19, 1.96)[c]	1.11 (1.02, 1.27)[bc]	129.06	<0.001
Tritriacontadiene* (53)	0.38 (0.35, 0.42)[a]	0.43 (0.39, 0.49)[a]	0.78 (0.61, 0.92)[b]	0.90 (0.66, 1.17)[b]	0.68 (0.61, 0.78)[b]	101.15	<0.001
Pentatriacontadiene* (61)	0.18 (0.15, 0.23)[a]	0.20 (0.17, 0.23)[a]	0.39 (0.27, 0.49)[b]	0.64 (0.37, 1.06)[b]	0.42 (0.31, 0.52)[b]	65.60	<0.001
Methylated alkanes							
11-Methylpentacosane, 9-Methylpentacosane (12)	0.00 (0.00, 0.01)[a]	0.01 (0.00, 0.01)[a]	0.02 (0.02, 0.03)[b]	0.02 (0.01, 0.03)[b]	0.01 (0.00, 0.01)[a]	95.86	<0.001
5-Methylpentacosane (13)	0.01 (0.01, 0.02)[abc]	0.01 (0.01, 0.02)[a]	0.02 (0.01, 0.03)[a]	0.02 (0.01, 0.02)[b]	0.01 (0.01, 0.01)[c]	29.84	<0.001
13-Methylheptacosane (22)	0.01 (0.01, 0.02)[a]	0.01 (0.01, 0.01)[b]	0.01 (0.01, 0.02)[ab]	0.01 (0.01, 0.02)[a]	0.02 (0.01, 0.02)[a]	29.56	<0.001
11-Methylheptacosane (23)	0.03 (0.02, 0.03)[a]	0.03 (0.02, 0.03)[a]	0.02 (0.01, 0.02)[b]	0.02 (0.02, 0.04)[a]	0.03 (0.03, 0.03)[a]	27.40	<0.001
3-Methylheptacosane (24)	0.03 (0.02, 0.03)[a]	0.03 (0.02, 0.03)[a]	0.07 (0.04, 0.09)[b]	0.03 (0.02, 0.04)[a]	0.03 (0.02, 0.03)[a]	55.40	<0.001

Continued on next page.

Compounds	Sp wax (*n* = 40)	Cp wax (*n* = 40)	Sterile worker (*n* = 31)	Fertile worker (*n* = 50)	Queen (*n* = 9)	H_4	*p*
Ethylesters							
Ethyl hexadecanoate (1)	0.44 (0.23, 0.61)[a]	0.24 (0.14, 0.36)[a]	0.14 (0.05, 0.24)[b]	0.22 (0.11, 0.31)[ab]	0.15 (0.14, 0.25)[a]	30.55	<0.001
Ethyl octacosanoate (50)	0.01 (0.00, 0.02)[a]	0.01 (0.01, 0.02)[a]	0.01 (0.07, 0.13)[b]	0.09 (0.06, 0.11)[b]	0.00 (0.00, 0.01)[a]	98.29	<0.001
Ethyl triacontanoate (60)	0.28 (0.24, 0.32)[a]	0.23 (0.19, 0.25)[ab]	0.05 (0.04, 0.10)[c]	0.13 (0.10, 0.16)[c]	0.15 (0.13, 0.17)[bc]	113.12	<0.001
Wax esters							
Hexadecyl tetradecanoate (49)	0.07 (0.06, 0.09)[a]	0.10 (0.08, 0.13)[b]	0.17 (0.12, 0.21)[b]	0.11 (0.06, 0.14)[b]	0.11 (0.08, 0.11)[ab]	43.70	<0.001
Icosyl hexadecanoate (66)	0.07 (0.05, 0.09)[a]	0.06 (0.05, 0.07)[a]	0.05 (0.03, 0.05)[a]	0.04 (0.03, 0.06)[a]	0.06 (0.04, 0.12)[a]	26.27	<0.001
Icosyl octadecenoate* (67)	0.60 (0.47, 0.81)[a]	0.58 (0.47, 0.90)[a]	0.85 (0.68, 1.45)[b]	0.46 (0.32, 0.60)[c]	0.12 (0.10, 0.15)[d]	59.29	<0.001
Docosyl hexadecanoate, Icosyl octadecanoate (68)	0.12 (0.10, 0.15)[a]	0.12 (0.10, 0.17)[a]	0.12 (0.08, 0.21)[a]	0.05 (0.03, 0.07)[b]	0.00 (0.00, 0.00)[b]	78.59	<0.001
Docosyl octadecenoate* (69)	0.46 (0.41, 0.53)[a]	0.40 (0.33, 0.54)[a]	0.45 (0.36, 0.72)[a]	0.31 (0.26, 0.44)[b]	0.18 (0.16, 0.21)[c]	52.31	<0.001
Tetracosyl hexadecanoate, Docosyl octadecanoate (70)	0.29 (0.24, 0.36)[a]	0.27 (0.23, 0.32)[a]	0.12 (0.09, 0.16)[b]	0.06 (0.05, 0.10)[b]	0.02 (0.00, 0.04)[b]	101.00	<0.001
Tetracosyl octadecenoate* (71)	4.35 (4.23, 4.55)[a]	4.49 (4.36, 4.67)[a]	3.49 (2.18, 4.60)[a]	4.36 (3.77, 5.22)[a]	5.21 (3.83, 5.90)[a]	20.43	<0.001
Hexacosyl hexadecanoate, Tetracosyl octadecanoate (72)	0.40 (0.33, 0.48)[a]	0.40 (0.34, 0.47)[a]	0.18 (0.13, 0.22)[b]	0.13 (0.11, 0.18)[b]	0.07 (0.07, 0.10)[b]	109.35	<0.001
Hexacosyl octadecenoate* (73)	11.07 (10.60, 11.60)[a]	11.86 (11.53, 12.40)[a]	5.49 (3.24, 8.02)[b]	8.32 (7.68, 9.52)[b]	7.67 (7.07, 8.76)[b]	125.88	<0.001
Octacosyl hexadecanoate, Hexacosyl octadecanoate (74)	0.51 (0.41, 0.60)[a]	0.45 (0.38, 0.51)[a]	0.22 (0.16, 0.29)[b]	0.28 (0.19, 0.35)[b]	0.14 (0.12, 0.15)[b]	99.64	<0.001
Octacosyl octadecenoate* (75)	2.27 (2.01, 2.46)[a]	2.37 (2.17, 2.58)[a]	0.97 (0.47, 1.39)[b]	1.43 (1.24, 1.62)[b]	1.41 (1.25, 1.52)[b]	124.20	<0.001
Triacontyl octadecenoate* (76)	3.69 (2.95, 3.98)[a]	3.41 (2.88, 4.11)[a]	0.95 (0.51, 1.73)[b]	1.94 (1.51, 2.26)[bc]	2.40 (2.26, 2.83)[ac]	111.11	<0.001
Aldehydes							
Triacontanal (58)	0.50 (0.43, 0.58)[a]	0.49 (0.37, 0.58)[a]	0.25 (0.13, 0.46)[b]	0.54 (0.38, 0.62)[a]	0.41 (0.37, 0.49)[a]	23.06	<0.001
Dotriacontanal (65)	0.69 (0.54, 0.76)[a]	0.53 (0.40, 0.70)[ab]	0.20 (0.05, 0.36)[c]	0.44 (0.29, 0.62)[b]	0.65 (0.45, 0.69)[ab]	53.93	<0.001
Ketones							
2-Nonacosanone (48)	0.15 (0.07, 0.25)[a]	0.10 (0.05, 0.14)[ab]	0.06 (0.03, 0.11)[b]	0.10 (0.07, 0.13)[ab]	0.07 (0.05, 0.10)[ab]	22.121	<0.001
2-Triacontanone (59)	0.04 (0.03, 0.06)[a]	0.08 (0.06, 0.11)[b]	0.07 (0.05, 0.09)[b]	0.08 (0.06, 0.10)[b]	0.03 (0.03, 0.05)[a]	38.14	<0.001

Continued on next page.

Compounds	Sp wax (*n* = 40)	Cp wax (*n* = 40)	Sterile worker (*n* = 31)	Fertile worker (*n* = 50)	Queen (*n* = 9)	H_4	*p*
Acetates							
Tetracosyl acetate (27)	0.02 (0.02, 0.03)[ab]	0.03 (0.02, 0.03)[a]	0.02 (0.01, 0.03)[bc]	0.01 (0.01, 0.02)[cd]	0.01 (0.01, 0.01)[d]	59.63	<0.001
Hexacosyl acetate (39)	0.06 (0.05, 0.07)[a]	0.06 (0.06, 0.08)[a]	0.10 (0.07, 0.14)[b]	0.06 (0.03, 0.07)[a]	0.01 (0.01, 0.01)[c]	61.82	<0.001
Unidentified							
Unidentified (15)	0.03 (0.01, 0.05)[a]	0.02 (0.02, 0.03)[a]	0.03 (0.02, 0.05)[a]	0.02 (0.02, 0.03)[a]	0.01 (0.01, 0.01)[b]	25.70	<0.001
Unidentified (20)	0.69 (0.63, 0.73)[a]	0.61 (0.55, 0.68)[ab]	0.53 (0.42, 0.68)[ab]	0.57 (0.42, 0.68)[b]	0.29 (0.25, 0.31)[c]	32.13	<0.001
Unidentified (46)	0.53 (0.46, 0.64)[a]	0.47 (0.36, 0.52)[b]	0.65 (0.45, 0.98)[a]	0.32 (0.22, 0.46)[b]	0.69 (0.54, 0.84)[a]	54.19	<0.001
Unidentified (57)	0.73 (0.66, 0.79)[a]	0.61 (0.51, 0.67)[a]	0.27 (0.14, 0.32)[b]	0.26 (0.20, 0.37)[b]	0.20 (0.18, 0.26)[b]	121.47	<0.001
Unidentified (62)	0.01 (0.01, 0.02)[a]	0.01 (0.01, 0.02)[a]	0.02 (0.02, 0.03)[b]	0.05 (0.03, 0.11)[b]	0.04 (0.04, 0.06)[b]	94.36	<0.001
Unidentified (64)	0.10 (0.08, 0.11)[a]	0.08 (0.06, 0.10)[ab]	0.05 (0.03, 0.10)[b]	0.10 (0.08, 0.13)[a]	0.07 (0.05, 0.08)[b]	26.58	<0.001

asterik: unknown double bond position
